# Mortality risk in patients with anti-MDA5 dermatomyositis is related to rapidly progressive interstitial lung disease and anti-Ro52 antibody

**DOI:** 10.1186/s13075-023-03100-z

**Published:** 2023-07-24

**Authors:** Huyan Wang, Xin Chen, Yan Du, Lihua Wang, Qiyuan Wang, Huaxiang Wu, Lei Liu, Jing Xue

**Affiliations:** 1 Department of Rheumatology, The Second Affiliated Hospital of Zhejiang University School of Medicine, Jiefang Road, Hangzhou, 310000 China; 2 Department of Rheumatology, The First Affiliated Hospital of Ningbo University, Ningbo, China; 3 Department of Radiology, The Second Affiliated Hospital of Zhejiang University School of Medicine, Hangzhou, China

**Keywords:** Dermatomyositis, Anti-MDA5 antibody, Anti-Ro52 antibody, Rapidly progressive interstitial lung disease, Prognosis

## Abstract

**Background:**

This study aimed to describe the clinical characteristics and analyze the poor prognostic factors in patients with anti-MDA5 dermatomyositis.

**Methods:**

A total of 126 adults with anti-MDA5 dermatomyositis were enrolled in this retrospective study. Information on survival time, cause of death, and baseline characteristics was collected. Patients were divided into two groups: a survival group and a non-survival group. Items with clinical significance that showed significant differences between the two groups were screened by Kaplan–Meier and Cox regression analyses to identify the predictors of poor survival.

**Results:**

Thirty-two patients were included in the non-survival group, most of whom died from respiratory failure, with pulmonary infection accounting for half. Epstein–Barr virus infection was relatively common in both groups. Aspartate transaminase, lactate dehydrogenase, and ferritin levels; erythrocyte sedimentation rate; and anti-Ro52 antibody levels were significantly higher, while the lymphocyte count was lower in the non-survival group compared with the survival group. Notably, patients in the non-survival group were more likely to present with rapidly progressive interstitial lung disease than those in the survival group. Kaplan–Meier and Cox multivariate regression analyses revealed that the prevalence of rapidly progressive interstitial lung disease, levels of anti-Ro52 antibody, and age > 57 years were important prognostic factors independent of multiple clinical parameters.

**Conclusions:**

Rapidly progressive interstitial lung disease, anti-Ro52 antibody levels, and age > 57 years are possible predictors of mortality risk in patients with anti-MDA5 dermatomyositis.

**Supplementary Information:**

The online version contains supplementary material available at 10.1186/s13075-023-03100-z.

## Background

Dermatomyositis (DM) is a systemic, heterogeneous disease that presents with skeletal muscle weakness, typical skin lesions, and other internal organ damage, usually with specific autoantibodies. The autoantibody against melanoma differentiation-associated gene 5 (anti-MDA5ab), first discovered by Sato et al. in 2005, has attracted extensive attention due to its association with the specific form of DM known as amyopathy or hypomyopathy [1].

Interstitial lung disease (ILD) is one of the most frequent and serious manifestations of MDA5+DM, with a much higher prevalence of 50–100% compared with other types of DM [2, 3]. The precise pathogenesis of ILD in patients with MDA5+DM remains unclear, although some evidence suggests that pulmonary vasculopathy, anti-MDA5ab-mediated endothelial cell injury, and increased expression of pro-fibrogenic cytokines may be involved [4–6]. Rapidly progressive ILD (RP-ILD) is a refractory and devastating complication and arguably a major cause of death in patients with this disease. In addition, several studies showed that serum ferritin and other cytokine profiles were correlated with the clinical course and complications of MDA5+DM. However, the differing clinical features of MDA5+DM among patients mean that it is hard to clarify disease severity based on a single factor. Some studies have identified phenotypic variations of MDA5+DM by cluster analysis, with patients in different clusters having highly heterogenic manifestations and outcomes. Phenotypes characterized by rashes, arthritis, skin vasculopathy, and serum hypoinflammatory status had a lower risk of death, while phenotypes involving severe pulmonary manifestations were always linked to poor outcomes [7–9].

Patients with autoimmune diseases are particularly vulnerable to infection, possibly because of disorders in the intrinsic immune environment, secondary immunodeficiency due to the use of immunosuppressants, and underlying complications. The timely diagnosis and treatment of infections is critical for the clinical prognosis of these patients.

The current study aimed to investigate and compare the clinical characteristics of patients with MDA5+DM who survived and those who died, to identify potential prognostic predictors in a Chinese cohort of patients with MDA5+DM.

## Methods

### Inclusion criteria and ethics

This retrospective study included 126 consecutive patients diagnosed with MDA5+DM who were followed up in the Department of Rheumatology, the Second Affiliated Hospital of Zhejiang University School of Medicine, China, from February 2017 to October 2021. The diagnosis of MDA5+DM was based on the criteria of the 239th ENMC International Workshop [10] as positive anti-MDA5ab and typical DM rashes. Myositis-specific autoantibodies (MSAs) and myositis-associated autoantibodies (MAAs) against MDA5, Jo‐1, OJ, EJ, PL‐7, PL‐12, Mi‐2α, Mi‐2β, TIF1γ, SAE1, NXP2, SRP, Ku, PM‐Scl75, PM‐Scl100, and Ro‐52 were detected using a commercial immunoblot assay (Autoimmune Inflammatory Myopathies 16 Ag-(IgG); EUROLINE, Lübeck, Germany). The results were defined as negative for greyscale values of 0–10 U/L; weakly positive, 11–25 U/L; moderately positive, 26–50 U/L; and strongly positive, > 50 U/L. The study was approved by the Ethics Committee of the Second Affiliated Hospital of Zhejiang University School of Medicine in China [No. 2018 (222)]. The ethics committee approved the waiver of informed consent due to the retrospective nature of the study.

### Clinical data

Thirty-two patients had died, and 94 were still alive up to January 2022, as confirmed by a telephone follow-up survey. Clinical data including complete medical histories and laboratory and physical examinations were obtained at first admission. Each patient underwent high-resolution chest computed tomography (HRCT). Pulmonary function measurements were obtained for 79 patients. Microbiologic testing was also carried out to detect infections, as clinically indicated, including Gram staining, bacterial or fungal smears and cultures, Epstein–Barr virus (EBV) and cytomegalovirus IgM and IgG antibody tests and nucleic acid detection, (1,3)-β-D-glucan assays and galactomannan tests, *Cryptococcus neoformans* capsular polysaccharide antigen detection, *Aspergillus* IgG antibody detection, and respiratory microorganism IgM antibody testing.

### Definitions

ILD was established by the presence of specific chest-imaging features, such as reticular pattern, ground-glass opacities, consolidations, honeycombing, or traction bronchiectasis displayed in HRCT scanned by two independent radiologists. RP-ILD was defined as acute and progressive ILD in patients who developed dyspnea and worsening interstitial changes on chest HRCT within 3 months since the onset of respiratory symptoms [2]. Chronic ILD (C-ILD) was ILD that did not fulfill the criteria for RP-ILD. DM-related malignancies were malignancies that had been clearly diagnosed from 3 years before the diagnosis of DM to the last follow-up. “Glucocorticoids only” represented patients who were treated with glucocorticoids (GCs) but without other conventional synthetic disease-modifying antirheumatic drugs (DMARDs). “Dual therapy” referred to traditional empirical therapy, namely GCs combined with one kind of DMARDs (e.g., cyclophosphamide, tacrolimus, cyclosporine A, mycophenolate mofetil, thalidomide), and “triple therapy” was a Japanese protocol combining concurrent high-dose GCs, tacrolimus, and intravenous cyclophosphamide.

### Statistical analysis

The median values were compared using the nonparametric Mann–Whitney *U* test and mean values using Student’s *t*-test. Frequencies were compared using *χ* ^2^ or Fisher’s exact test. Optimal grouping cutoff points for continuous data associated with prognosis were identified using the X-tile software [11]. The cumulative survival rate was calculated by Kaplan–Meier curve analysis, and log-rank tests were used to examine the differences in survival curves among the variables. Risk factors related to prognosis were determined by univariate and multivariate Cox proportional hazards regression models, with variables with *p* < 0.05 in univariate analysis selected for multivariate analysis. A *p* value < 0.05 was considered statistically significant. Statistical analysis and graphical representation were conducted using SPSS v. 25.0 and GraphPad Prism v. 8.0, respectively.

## Results

### Demographic characteristics and follow-up

The patient characteristics are shown in Table 1. Patients in the non-survival group were significantly older than those in the survival group, while the survivors included significantly more females than the non-survivors. The median time from the onset of symptoms to diagnosis was shorter in the non-survival group. The median follow-up time for all patients by January 2022 was 25.2 [11.1, 39.7] months (calculated by SPSS); however, the median survival time could not be estimated directly because the censoring proportion was > 50%. We therefore presented the actual median survival time, which was significantly shorter in the non-survival group compared with the survival group (3.6 months vs. 30.3 months, *p* < 0.001).

**Table 1:** Comparison of demographics, clinical and laboratory data, primary pulmonary function parameters, and ILD subtypes in patients with MDA5+DM between the survival and non-survival groups

	Non-survival group (*N* = 32)	Survival group (*N* = 94)	*P*-value
Female, no. (%)	14 (43.8)	64 (68.1)	**0.014**
Age at onset, years, median [IQR]	59 [55, 56]	48 [39, 57]	**< 0.001**
Diagnosis time, months, median [IQR]	1.0 [1.0, 3.0]	2.0 [1.0, 5.0]	**0.036**
Survival time, months, median [IQR]	3.6 [1.7, 7.2]	30.3 [15.0, 46.3]	**< 0.001**
Cause of death, no. (%)			
Respiratory failure	30 (93.8)	–	–
Gastrointestinal hemorrhage	1 (3.1)	–	–
Unknown	1 (3.1)	–	–
Laboratory data, median [IQR]			
ALT, U/L	71 [30, 133]	44 [24, 88]	0.069
AST, U/L	76 [48, 153]	46 [29, 87]	**0.008**
LDH, U/L	372 [284, 478]	301 [237, 368]	**0.008**
CK, U/L	85 [43, 276]	60 [36, 103]	0.079
Creatinine, μmol/L	56 [41, 60]	51 [41, 60]	0.385
Ferritin, ng/mL	1301 [523, 1500]	532 [241, 1215]	**0.003**
CRP, mg/L	14 [6, 25]	6 [3, 14]	**0.002**
ESR, mm/h	38 [28, 69]	28 [19, 47]	**0.011**
Total lymphocyte count, /µL	605 [390, 785]	745 [500, 1198]	**0.030**
CD3+T lymphocyte count, /µL	362 [254, 527]	454 [292, 759]	**0.025**
CD4+T lymphocyte count, /µL	249 [118, 385]	249 [162, 444]	0.241
CD8+T lymphocyte count, /µL	119 [66, 150]	145 [83, 253]	**0.029**
Clinical characteristics, no. (%)			
Myodynia	10 (31.3)	27 (28.7)	0.786
Muscle weakness	9 (28.1)	26 (27.7)	0.960
Heliotropic rash	19 (59.4)	57 (60.6)	0.900
V sign	12 (37.5)	42 (44.7)	0.478
Shawl sign	9 (28.1)	23 (24.5)	0.681
Gottron’s sign	25 (78.1)	75 (79.8)	0.841
Skin ulcer	5 (15.6)	9 (9.6)	0.347
Mechanics hand	10 (31.3)	33 (35.1)	0.691
Periungual erythema	8 (25.0)	23 (24.5)	0.952
Dysphagia	3 (9.4)	7 (7.4)	1.000
Malignancy	3 (9.4)	3 (9.4)	0.348
Overlap CTDs	0 (0)	7 (7.4)	0.190
Pulmonary function			
FVC, % pred, mean ± SD	66 ± 21^a^	75 ± 19^b^	0.091
FEV1, % pred, mean ± SD	66 ± 19^a^	74 ± 18^b^	0.093
DLco, % pred, mean ± SD	49 ± 33^c^	56 ± 17^d^	0.489
TLC, % pred, median [IQR]	67 [56, 78]^c^	75 [64, 86]^d^	0.213
ILD subtypes, no. (%)			
RP-ILD	29 (90.6)	9 (9.6)	**< 0.001**
C-ILD	2 (6.3)	79 (84.0)	**< 0.001**
Non-ILD	1 (3.2)	6 (6.4)	0.804

Bold text highlights the significance

*MDA5+DM* Anti-MDA5 dermatomyositis, *ALT* Alanine transaminase, *AST* Aspartate transaminase, *LDH* Lactate dehydrogenase, *CK* Creatine kinase, *CRP* C-reactive protein, *ESR* Erythrocyte sedimentation rate, *CTDs* Connective tissue diseases, *FVC* forced vital capacity, *FEV1* Forced expiratory volume in 1 s, *Dlco*, diffusing capacity of the lungs for carbon monoxide, *TLC* Total lung capacity, *% pred* Percent predicted values, *RP-ILD* Rapidly progressive interstitial lung disease, *C-ILD* Chronic interstitial lung disease

^a^Data available for 17 patients

^b^Data available for 62 patients

^c^Data available for 14 patients

^d^Data available for 61 patients

### Incidence of mortality and death pattern

Thirty-two of the 126 patients with MDA5+DM had died by the end of follow-up, giving a mortality rate of 25.4%. Of these patients who died, 30 had respiratory failure and all of these had ILD, complicated by pulmonary infection in 50% of cases. One patient died of gastrointestinal hemorrhage, and the remaining patient died from an unidentified cause (Table 1). Among the deaths, 37.5% and 68.8% occurred during the first 3 and 6 months after disease onset, respectively. The 3- and 6-month cumulative survival rates of the patients with MDA5+DM were 90% and 82%, respectively.

### Infection sites and pathogens identified by microbiologic testing

**Fig. 1:**
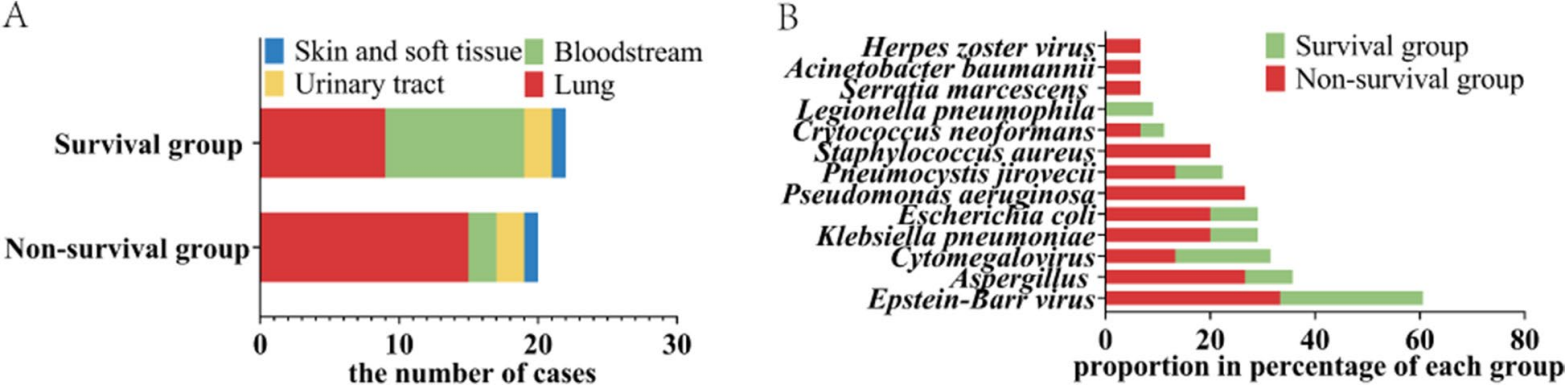
The main sites of infection. Common pathogens in the survival and non-survival groups

The incidence of infections was significantly higher in the non-survival group than in the survival group [46.9% (15/32) vs. 23.4% (22/94), *p* = 0.012]. The main sites of infection were the lung and bloodstream (Fig. 1A). Four patients in the non-survival group had multiple sites of infection, including one with simultaneous lung, bloodstream, and skin infections, one with lung and bloodstream infections, and two with lung and urinary tract infections. The top three pathogens identified in the non-survival group were EBV (*n* = 5), *Aspergillus* (*n* = 4), and *Pseudomonas aeruginosa* (*n* = 4), while EBV (*n* = 6) and cytomegalovirus (*n* = 4) were the most common pathogens in the survival group (Fig. 1B).

### Clinical profiles

Regarding laboratory examinations, levels of aspartate transaminase (AST), lactate dehydrogenase (LDH), ferritin, and C-reactive protein (CRP), and erythrocyte sedimentation rate (ESR) were significantly higher, while total lymphocyte, CD3+ T lymphocyte, and CD8+ T lymphocyte counts were lower in the non-survival group compared with the survival group (Table 1).

There were no significant differences in clinical manifestations including dysphagia and skin and muscle symptoms between the two groups (Table 1). Some patients were complicated with malignancies, including three cases of thyroid cancer in the survival group and two cases of bladder cancer and one of thyroid cancer in the non-survival group. Seven patients in the survival group had other connective tissue diseases with varying anti-MDA5ab levels, including two cases of Sjögren’s syndrome (3+, 2+), two cases of systemic lupus erythematosus (3+, 1+), two cases of rheumatoid arthritis (3+, 3+), and one case of ankylosing spondylitis (3+).

The baseline pulmonary function results are shown in Table 1. Data were lacking for some critical patients who could not complete the examinations due to dyspnea and weakness. The percent predicted forced vital capacity, forced expiratory volume in 1 s, diffusing capacity of the lungs for carbon monoxide, and total lung capacity values were all lower in the non-survival group compared with the survival group, although the differences were not significant.

The frequency of RP-ILD was significantly higher in the non-survival group compared with the survival group [90.6% (29/32) vs. 9.6% (9/94), *p* < 0.001]. Conversely, C-ILD was more common in the survival group. In addition, a small minority of patients (1 in the non-survival group and 2 in the survival group) had no lung involvement.

There was no difference in the proportions of patients with different anti-MDA5ab levels between the two groups (Table 2). In addition to the anti-MDA5 antibody, coexisting MSAs were found in two subjects in the survival group, including one with anti-TIF-1γ+++ and one with anti-SRP+++. Regarding MAAs, anti-Ro52 antibody (anti-Ro52ab) was the most common type and was isolated from 76/126 patients and was significantly more frequent in the non-survival compared with the survival group [81.3% (26/32) vs. 53.2% (50/94), *p* = 0.005]. The patients were divided into three subgroups according to anti-Ro52ab levels. Significantly more patients in the non-survival group were strongly positive for anti-Ro52ab compared with the survival group, but there was no clear difference in terms of the weakly positive group (Table 2). Other, less-frequent MAAs were found in four patients, including one non-survivor (PM-Scl75+) and three survivors (anti-PM-Scl75+, anti-PM-Scl100+, anti-Ku+).

**Table 2:** Comparison of anti-MDA5 and anti-Ro52 antibody levels between patients with MDA5+DM in the survival and non-survival groups

	Non-survival group (*N* = 32)	Survival group (*N* = 94)	*P*-value
MDA5, no. (%)			
Weak (1+)	2 (6.3)	11 (11.7)	0.590
Moderate (2+)	3 (9.4)	12 (12.8)	0.845
Strong (3+)	27 (84.4)	71 (75.5)	0.299
Ro52, no. (%)			
Negative	6 (18.8)	44 (46.8)	**0.005**
Weak (1+)	1 (3.1)	11 (11.7)	0.281
Moderate (2+)	4 (12.5)	11 (11.7)	1.000
Strong (3+)	21 (65.6)	28 (29.8)	**< 0.001**

Data presented as number and percentage. Bold text highlights the significance

*MDA5+DM*, anti-MDA5 dermatomyositis

There were several differences in the medications received between the two groups (Table 3). More patients with adverse outcomes had received intravenous pulse methylprednisolone and intravenous immunoglobulin during their lifetime. However, there were no major differences in the use of biological agents or immunosuppressants between the two groups, whether treated with dual therapy or triple therapy.

**Table 3:** Comparison of medications between patients with MDA5+DM in the survival and non-survival groups

	Non-survival group (*N* = 32)	Survival group (*N* = 94)	*P*-value
Glucocorticoids only	12 (37.5)	16 (17.0)	**0.016**
Dual therapy	16 (50.0)	58 (61.7)	0.245
Glucocorticoids + tacrolimus	5 (15.6)	31 (33.0)	0.061
Glucocorticoids + intravenous cyclophosphamide	7 (21.9)	24 (25.5)	0.678
Glucocorticoids + mycophenolate mofetil	1 (3.1)	1 (1.1)	1.000
Glucocorticoids + cyclosporine A	2 (5.9)	2 (2.1)	0.615
Glucocorticoids + thalidomide	1 (3.1)	0 (0)	0.254
Triple therapy	4 (12.5)	20 (21.3)	0.275
Intravenous pulse methylprednisolone	7 (21.9)	6 (6.4)	**0.013**
Rituximab	2 (6.3)	1 (1.1)	0.322
Tocilizumab	2 (6.3)	1 (1.1)	0.322
Tofacitinib	0 (0)	9 (9.6)	0.110
Intravenous immunoglobulin	21 (65.5)	40 (42.6)	**0.024**

Data presented as number and percentage. Pulse methylprednisolone includes treatment with glucocorticoids alone or glucocorticoids with other immunosuppressants. Triple therapy includes high-dose glucocorticoids, tacrolimus, and intravenous cyclophosphamide. Bold text highlights the significance

*MDA5+DM*, anti-MDA5 dermatomyositis

### Kaplan–Meier survival curves of determinant variables

The cutoff points of the variables that significantly increased the incidence of death, calculated by the X-tile software, were age > 57 years, ferritin > 1002.6 ng/mL, LDH > 388 U/L, and ESR > 63 mm/h (Additional file 1: Fig. S1). However, the total lymphocyte count, CD3+ T lymphocyte count, and CD8+ T lymphocyte count could not be converted into categorical variables using the X-tile software to determine their impacts on survival.

**Fig. 2:**
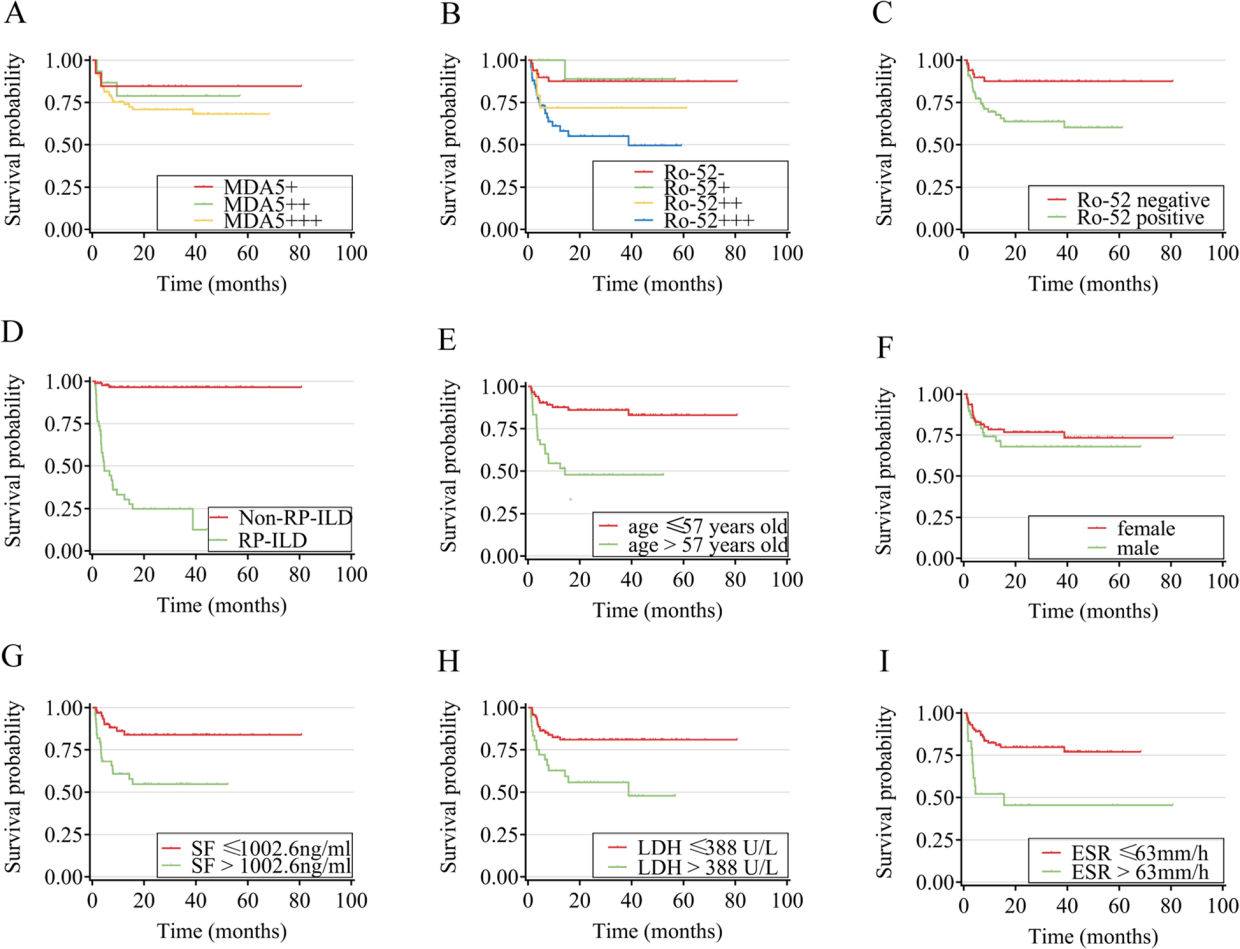
Cumulative survival probability

Survival time was largely unaffected by the anti-MDA5ab level. In contrast, patients positive for anti-Ro52ab tended to have shorter survival times than those negative for anti-Ro52ab (log-rank *p* = 0.006). Moreover, patients strongly positive (3+) for anti-Ro52ab had a worse prognosis than those who were antibody-negative or weakly positive (1+) (log-rank *p* = 0.001 and 0.029, respectively). Patients without RP-ILD had longer survival times than those with RP-ILD, whose median survival time was 4.5 months (log-rank *p* < 0.001). Regarding age, patients ≤ 57 years old had longer survival times than those > 57 years old (log-rank* p* < 0.001). Females appeared to have a longer survival time than males, but the difference was not significant. Lastly, ferritin > 1002.6 ng/mL (log-rank* p* < 0.001), LDH > 388 U/L (log-rank* p* = 0.002), and ESR > 63 mm/h (log-rank* p* < 0.001) were significantly associated with shorter survival times (Fig. 2).

### Univariate and multivariate cox regression analyses

The Cox regression model for the eligibility of the proportional hazard assumptions was checked using the Schoenfeld residuals test for continuity variables, which confirmed that the assumption was met (*p* > 0.05).

The results of univariate and multivariate Cox regression analyses for the effects of each prognostic factor on the survival of patients with MDA5+DM are presented in Table 4. Univariate regression analysis identified female sex, age > 57 years, ferritin > 1002.6 ng/mL, LDH > 388 U/L, ESR > 63 mm/h, CD3+ T lymphocyte count, CD8+ T lymphocyte count, anti-Ro52ab level, and RP-ILD as significant factors (*p* < 0.05), and these variables were therefore included as covariates in the multivariate regression analysis. Multivariate Cox regression analysis showed that the hazard ratio (HR) for death among patients with RP-ILD was 25.07-fold (95% confidence interval (CI): 5.42–115.98) higher compared with patients without RP-ILD. Anti-Ro52 antibody levels were also an independent determinant of time to death (HR 3.41, 95% CI: 1.36–8.53), and age > 57 years was significantly associated with the risk of death (HR 4.45, 95% CI: 2.17–9.15).

**Table 4:** Univariate and multivariate Cox proportional hazards analyses of risk factors for death in patients with MDA5+DM

Variable	Univariate		Multivariate	
	HR (95% CI)	*P*-value	HR (95% CI)	*P*-value
Sex, female	0.45 (0.22–0.90)	**0.024**	0.75 (0.31–1.78)	0.512
Age > 57 years old	4.45 (2.17–9.15)	**< 0.001**	3.05 (1.20–7.80)	**0.020**
Ferritin > 1002.6 ng/mL	3.54 (1.60–7.82)	**0.002**	0.89 (0.29–2.76)	0.837
LDH > 388 U/L	2.92 (1.46–5.85)	**0.002**	1.29 (0.39–4.22)	0.674
ESR > 63 mm/h	3.54 (1.72–7.28)	**0.001**	1.78 (0.73–4.38)	0.208
Total lymphocyte count (/µL)	1.00 (1.00–1.00)	0.071	–	–
CD3+T lymphocyte count (/µL)	1.00 (1.00–1.00)	**0.021**	1.00 (1.00–1.00)	0.828
CD8+T lymphocyte count (/µL)	1.00 (0.99–1.00)	**0.023**	1.00 (0.99–1.01)	0.978
Levels of anti-MDA5 antibody	1.43 (0.75–2.71)	0.277	–	–
Levels of anti-Ro52 antibody	1.70 (1.26–2.31)	**0.001**	3.41 (1.36–8.53)	**0.009**
RP-ILD	37.73 (11.34–125.6)	**< 0.001**	25.07 (5.42–115.98)	**< 0.001**

Bold text highlights the significance

“–” indicates not included in the multivariate model due to the lack of significant association in univariate analysis

*MDA5+DM* Anti-MDA5 dermatomyositis, *LDH* Lactate dehydrogenase, *ESR* Erythrocyte sedimentation rate, *RP-ILD* Rapidly progressive interstitial lung disease

## Discussion

Recent research on autoantibodies and clinical practice have produced new insights into the various subtypes of DM associated with different MSAs, which have in turn played important roles in determining the clinical features and assessing the prognosis of DM. MDA5+DM has been widely considered as a more severe subtype compared with other types of DM. In the current study, we sought to determine the risk factors influencing survival in patients with MDA5+DM.

Previous studies have highlighted the clinical features of the acute form of MDA5+DM; data available on the long-term clinical course and hazard factors is minimal. In China, many cohorts used the occurrence of RP-ILD as an endpoint in the studies of MDA5+DM prognosis [12–15]. However, we focus on comparing the data of the patients who deceased and the others who still survived. Mortality is an unequivocal end point which can directly measure deterioration. Long-term follow-up outcome makes it establish more clinically meaningful and reliable proxies for prognosis. For those studies in which death was the primary outcome, the sample size was also significantly smaller in other centers than in this report [16], except for retrospective multicenter cohorts [17, 18], and the follow-up time of some studies is shorter [19, 20].

The overall mortality in the current study was 25.4%, which was similar to several other studies (23.4–37.9%) [7, 9, 18]. Our data also supported previous studies showing that death was a time-dependent change and often occurred in the early stage of disease [21]. The high risk of death at this stage highlights the need to strengthen disease-progression monitoring.

The relationship between specific autoantibodies and disease activity or prognosis in idiopathic inflammatory myositis is currently unclear. Gono et al. [22] found that anti-MDA5ab titers were higher at admission and decreased less after treatment in non-survivors. In contrast, the current study found no significant difference in survival rates in relation to anti-MDA5ab levels at baseline, probably because subjects who received a diagnosis of MDA5+DM usually had high levels determined by semi-quantitative measurements, and differences may become evident if quantitative assays are used. Other studies showed that anti-MDA5ab levels tended to decline significantly at remission but increase again during relapse, suggesting that anti-MDA5ab could be used to evaluate disease activity and response to treatment [23, 24]. Unfortunately, the current study only evaluated anti-MDA5ab at a single time point and did not track the longitudinal serological changes.

Patients with MDA5+DM also exhibit a high prevalence of anti-Ro52ab. A previous study in a cohort of 267 patients with idiopathic inflammatory myositis-associated ILD found that anti-Ro52ab was more frequent in patients with anti-MDA5 and anti-Jo-1 antibodies than in those with other MSAs [25]. Xu et al. [16] showed that anti-Ro52ab was associated with a higher prevalence of RP-ILD in patients with anti-MDA5ab-positive clinically amyopathic dermatomyositis-ILD, while the cumulative 24-month survival rate was lower in patients with anti-Ro52ab than in those without. The current study further demonstrated that patients with MDA5+DM who were strongly positive for anti-Ro52ab tended to have a poorer prognosis. These results suggest that patients with MDA5+DM and anti-Ro52ab are more likely to have a high-risk clinical phenotype, although the cause remains unclear. The presence of anti-Ro52ab may reflect specific subtypes of this disease, and further stratification of patients with MDA5+DM based on the combination of anti-MDA5ab and anti-Ro52ab levels may help to provide more precise treatments and improve the long-term survival of patients.

Lymphocyte subsets play divergent roles in the cell microenvironment to maintain immune homeostasis. Blood lymphocyte counts, both CD4+ and CD8+ T lymphocytes, were significantly lower in patients with MDA5+DM compared with controls, especially in cases with aggravation of interstitial lung lesions [12, 26]. The current study enrolled patients who were diagnosed and treated for the first time and the use of immunosuppressants was therefore not considered to be responsible for the elimination of immunocytes. Mueller et al. [27] previously reported that effector memory T cells broadly migrated between peripheral nonlymphoid tissues (e.g., skin and lung), the circulation, and the spleen and emphasized potential differences in the migratory patterns between CD4+ and CD8+ T lymphocytes. In addition, non-recirculating tissue-resident memory T cells could localize within epithelial layers in previously challenged skin and lung airways. Further experiments are needed to determine the detailed mechanism.

The aggravation of primary lung lesions, deterioration caused by excessive immunosuppression, and secondary infection are currently recognized as the main causes of death in patients with MDA5+DM. The current study showed that patients who died of respiratory failure all had a complication of ILD and half also had lung infections. Our study confirmed that RP-ILD was significantly associated with poor survival rates and was the most critical predictor of mortality, with an almost 25-fold increased risk of death. Regarding lung function, all parameters were worse in the non-survival compared with the survival group, but the differences were not significant. This lack of significance may have been attributable to incomplete data.

The clinical management of MDA5+DM is challenging; no standard treatment has yet been established and current treatments are primarily empirical. Until Tsuji et al. [28] proposed an intensive regimen of “triple therapy,” the 6-month survival rate of patients with MDA5+DM undergoing conventional therapy remained poor. In addition, Janus kinase inhibitors have demonstrated favorable efficacy and safety [29]. However, the current study did not include a large proportion of patients treated with triple therapy, which may result from the short survival time. The condition of critical patients is prone to progress rapidly in the early stage, and high-dose GCs alone are therefore the primary choice to induce remission; however, their effect is likely to be unsatisfactory, even during active treatment, and the short course of the disease may provide no opportunity to initiate subsequent therapy.

Patients with autoimmune diseases are often susceptible to various infections, possibly due to immune deficiencies. In the current study, infection events occurred more frequently in the non-survival group, and it could be that the dosages of GCs were excessively high since the disease was most active in the early stage. Previous studies showed that underlying lung disease such as ILD might provide an accessible environment for infection [30]. Lung infection was indeed the most among all types of infection in the non-survivors, all of whom stricken with a lung infection were complicated with RP-ILD, so that dual setbacks led to a great increase in mortality. Moreover, our study found that EBV was common, but many patients lacked symptoms of current acute infection. EBV is usually acquired silently in life span and carried thereafter as an asymptomatic infection; thus, it was uncertain if the infection happened before or after the start of DM. Decreased immune control and increased reactivation of EBV has been found to be a contributing factor in the development of autoimmune diseases [31, 32]. Advances regarding how EBV infection may interrelate with MDA5+DM deserve attention.

This study had several limitations. It was a retrospective study from a single institution and may thus have included potential bias. In addition, missing data for some items, such as pulmonary function, was unavoidable. However, the study had the advantage of including a relatively large cohort of subjects diagnosed with MDA5+DM, recruited from a tertiary medical center in China, all of whom underwent HRCT, and the outcome for each subject was determined over a sufficiently long follow-up period.

## Conclusions

Overall, we described the demographic and clinical characteristics of patients with MDA5+DM in relation to survival outcomes and evaluated the risk factors affecting survival. The results suggest that patients with MDA5+DM with co-existing RP-ILD, as a predictive factor for prognosis, should receive intensive follow-up. Anti-Ro52ab is highly prevalent in patients with MDA5+DM, and high levels are associated with a low survival rate. Enhancement of risk stratification by combining anti-Ro52ab levels and age may help to provide effective and targeted treatment and further improve the overall outcomes of patients with MDA5+DM.

## Supplementary Information

**Additional file 1: Supplementary Figure 1.**

## Data Availability

All data generated or analyzed during this study are included in this published article and supplementary information files.

## Abbreviations

DM: Dermatomyositis
anti-MDA5ab: Autoantibody against melanoma differentiation-associated gene 5
RP-ILD: Rapidly progressive interstitial lung disease
MDA5+DM: Anti-MDA5 dermatomyositis
MSA: Myositis-specific autoantibody
MAA: Myositis-associated autoantibody
HRCT: Chest high-resolution computed tomography
EBV: Epstein–Barr virus
C-ILD: Chronic interstitial lung disease
GC: Glucocorticoid
AST: Aspartate transaminase
LDH: Lactate dehydrogenase
CRP: C-reactive protein
ESR: Erythrocyte sedimentation rate
SLE: Systemic lupus erythematosus
anti-Ro52ab: Anti-Ro52 antibody
