# Supplementary figures and images for: Mortality risk in patients with anti-MDA5 dermatomyositis is related to rapidly progressive interstitial lung disease and anti-Ro52 antibody
Source: Arthritis Res Ther. 2023 Jul 24;25:127. doi: 10.1186/s13075-023-03100-z (PMC10367378; doi:10.1186/s13075-023-03100-z)

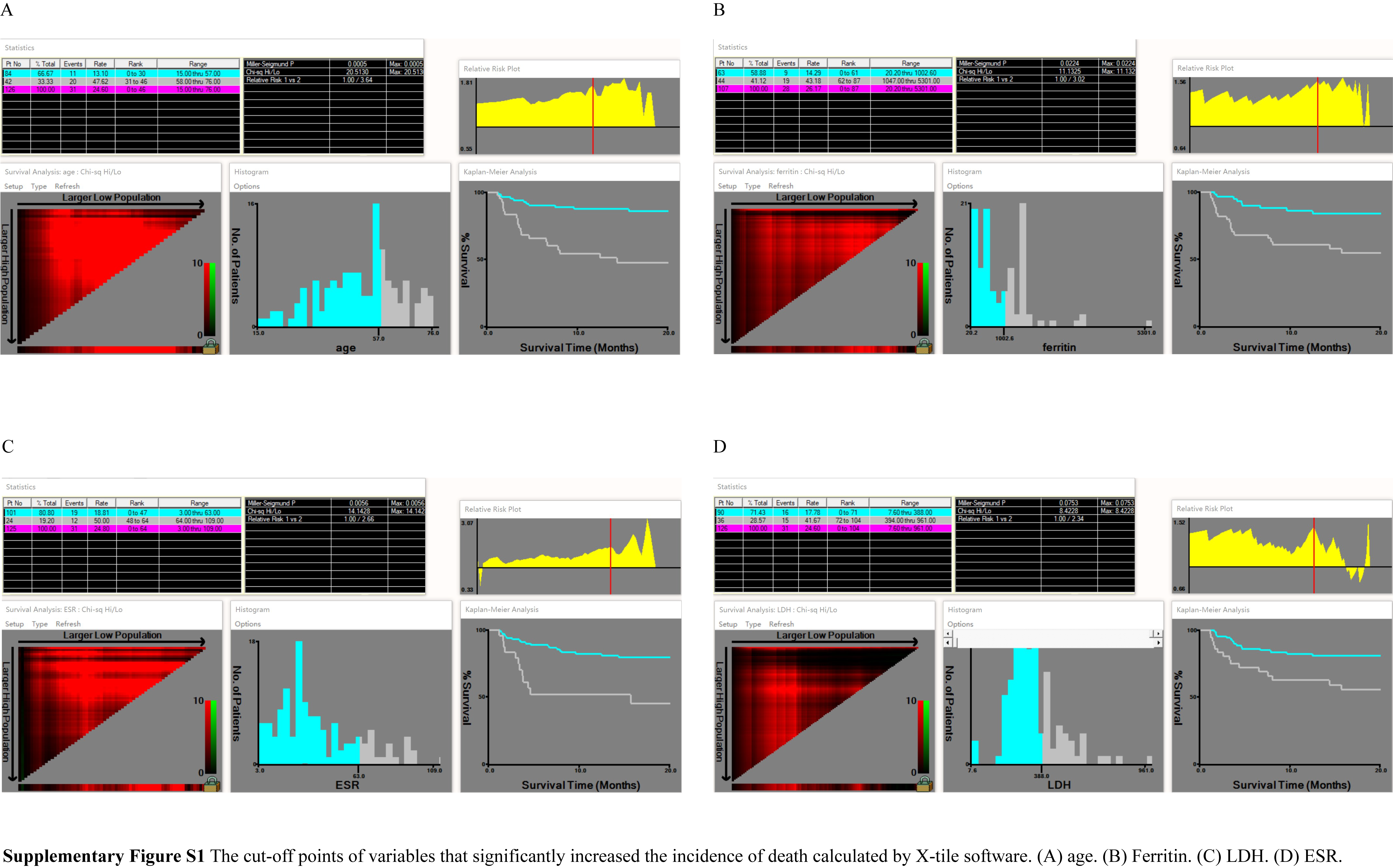

Supplement: Supplementary file 1 — Additional file 1: Supplementary Figure 1. [file 13075_2023_3100_MOESM1_ESM.tif]
